# Supplementary material for: Transcriptional Profiling of Malignant Melanoma Reveals Novel and Potentially Targetable Gene Fusions
Source: Cancers (Basel). 2022 Mar 15;14(6):1505. doi: 10.3390/cancers14061505 (PMC8946593; doi:10.3390/cancers14061505)
Supplement: Supplementary file 1 [file cancers-14-01505-s001.zip › cancers-1624799-supplementary.pdf]

Article

Transcriptional Profiling of Malignant Melanoma Reveals Novel and Potentially Targetable Gene Fusions

Sourat Darabi [1,*,†], Andrew Elliott [2,†], David R. Braxton [1], Jia Zeng [2], Kurt Hodges [2], Kelsey Poorman [2], Jeff Swensen [2], Basavaraja U. Shanthappa [2], James P. Hinton [2], Geoffrey T. Gibney [3], Justin Moser [4], Thuy Phung [5], Michael B. Atkins [3], Gino K. In [6], Wolfgang M. Korn [2], Burton L. Eisenberg [1,6] and Michael J. Demeure [1,7]

1 Hoag Family Cancer Institute, Newport Beach, CA 92663, USA; david.braxton@hoag.org (D.R.B.); burton.eisenberg@hoag.org (B.L.E.); michael.demeure@hoag.org (M.J.D.)
2 Caris Life Sciences, Phoenix, AZ 85040, USA; aelliott@carisls.com (A.E.); jzeng@carisls.com (J.Z.); khodges@carisls.com (K.H.); kpoorman@carisls.com (K.P.); jswensen@carisls.com (J.S.); usbasava@gmail.com (B.U.S.); jhinton@carisls.com (J.P.H.); wmkorn@carisls.com (W.M.K.)
3 Lombardi Comprehensive Cancer Center, MedStar Georgetown University Hospital, Washington, DC 20007, USA; geoffrey.t.gibney@gunet.georgetown.edu (G.T.G.); mba41@georgetown.edu (M.B.A.)
4 Honor Health Research Institute, Scottsdale, AZ 85258, USA; jmoser@honorhealth.com
5 Department of Pathology, University of South Alabama, Mobile, AL 36617, USA; tphung@health.southalabama.edu
6 Division of Oncology, Norris Comprehensive Cancer Center, University of Southern California, Los Angeles, CA 90033, USA; gino.in@med.usc.edu
7 Translational Genomics Research Institution, Phoenix, AZ 85004, USA
* Correspondence: sourat.darabi@hoag.org
† These authors contributed equally to this work.

Supplemental Table S1. Recurrent fusions with unknown oncogenicity. Fusions transcripts detected in ≥ 3 samples.

Gene Fusion	N events	Notes
RIPK1:SERPINB9	21	
LINC00910:RUVBL1	9	
MTAP:CDKN2B-AS1	8	Potential tumor suppressor inactivation
LYST:NID1	6	
CDK2:RAB5B	5	Melanoma-specific readthrough transcript
MRPS16:CFAP70	3	
PAPSS2:PTEN	3	Potential tumor suppressor inactivation
SDCCAG8:AKT3	3	
CTNNA1:MATR3	3	Readthrough transcript
KPNA2:BPTF	3	

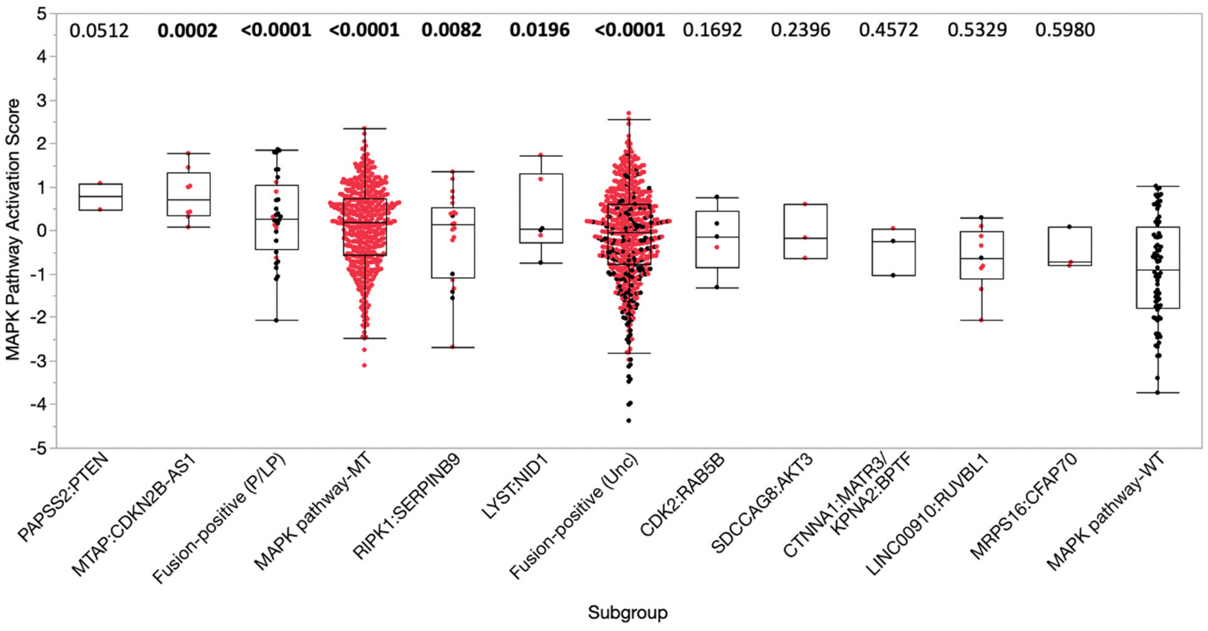

Supplemental Figure S1. MAPK pathway activation scores for recurrent fusions with unknown oncogenicity. Patient samples were stratified into subgroups based on the detection of unclassified fusion, with samples harboring a MAPK pathway alteration color-coded in red. P-values (Mann-Whitney U) noted above each subgroup reflect comparison with MAPK pathway-WT control subgroup.

Supplemental Table S2. Transcriptomic and immunohistochemical (IHC) analysis of MAPK pathway activation in fusion-positive melanoma. For 9 fusion-positive patient samples with available tissue, MAPK pathway activation was evaluated using antibodies to phospho-ERK1/2 (Thr202/Tyr204) and total-ERK1/2 protein, with the proportion of phosphorylated protein determined by the ratio of phospho:total-ERK1/2 H-scores (stain intensity * percentage of cells stained). Scores reflect fold-change of min-max normalized values. WT indicates no MAPK pathway alterations detected. WT and *BRAF*-MT scores represent the average of 3 independent samples.

			**Fold-change relative to WT**	
**Variant Subgroup**	**Variant**	**Co-alterations**	**Transcriptional MPAS score**	**Phospho:Total-ERK1/2 H-score**
WT	MAPK pathway-WT	PDL1+ (x2), *MDM2*-AMP (x1), *KIT*-AMP (x1)	1	1
BRAF-MT	*BRAF*-V600E	PDL1+ (x2)	3.44	2.17
BRAF Fusion	*ZSCAN25:BRAF*	----	3.29	5.35
	PDE4D:BRAF	*CDKN2A*-MT, *FBXW7*-MT	2.02	4.29
	CEP68:BRAF	*CDKN2A*-MT, *PTEN*-MT	2.80	2.09
	FLOT1:BRAF	----	4.24	0.87
RAF1 Fusion	*IMPDH2:RAF1*	TMB-H, *TP53*-MT, *CDH1*-MT	4.25	5.36
	PITPNA:RAF1	TMB-H, *BRCA1*-MT, *EZH2*-MT, *FBXW7*-MT	4.09	4.30
PRKCA Fusion	*NF1:PRKCA*	*NRAS*-MT	3.07	5.52
	ZNF814:PRKCA	*NF1*-MT, TMB-H, PDL1+, *ATM*-MT	3.29	4.66
	SEPT9:PRKCA	*NRAS*-MT, *NF1*-MT, *JAK1*-MT	3.12	2.17
